# Cloning and sequencing of the *ompL37* gene present in *Leptospira interrogans*, a surface protein in pathogenic leptospires

**Published:** 2019-10

**Authors:** Elaheh Rezaei, Pejvak Khaki, Soheila Moradi Bidhendi, Mojtaba Noofeli, Maryam Sadat Soltani

**Affiliations:** 1 Department of Microbiology, Razi Vaccine & Serum Research Institute, Agricultural Research, Education and Extension Organization (AREEO), Karaj, Iran; 2 Department of Human Bacterial Vaccines, Razi Vaccine & Serum Research Institute, Agricultural Research, Education and Extension Organization (AREEO), Karaj, Iran; 3 Department of Microbiology, Faculty of Biological Sciences, Shahid Beheshti University, Tehran, Iran

**Keywords:** Leptospirosis, Molecular cloning, *ompL37* gene

## Abstract

**Background and Objectives:**

Leptospirosis, an infection caused by pathogenic leptospires, is associated with insufficient sanitation and poverty. Leptospira is transmitted through contact with contaminated urine of reservoir animals. The primary objective of this study was to clone and sequence the *ompL37* gene present in local and vaccine serovars.

**Materials and Methods:**

A total of 16 *Leptospira interrogans* serovars were cultured in EMJH liquid medium. After growing, genomic DNA was extracted using phenol-chloroform method. Primer pair was synthesized to amplify the 996 bp *ompL37* sequence. The amplified *ompL37* gene was cloned into pTZ57R/T vector. The sequences obtained from this study were compared with an only recorded sequence in the Genbank by the Meg Align software.

**Results:**

PCR products showed an amplified 996bp *ompL37* gene product belonging to pathogenic serovars, while no *ompL37* products were amplified in non-pathogenic serovars. Sequences comparison tests from 16 native serotypes examined in this study displayed a similarity range of 84% to 99.5% among serovars used. The results showed that two serotypes of *L. interrogans* including Serjoehardjo (RTCC2810 and RTCC2821) had the highest identity up to 95.5%. Two serovars of *L. interrogans* including Pomona (RTCC2822) and Icterohaemorrhagiae (RTCC2823) had the lowest identity about 84%.

**Conclusion:**

As the results showed, *ompL37*, present on the surface of such bacteria, showed a conserved sequence. *ompL37*, as a key role in cell adhesion and pathogenicity, can be used for designing diagnostic tests and vaccines. Furthermore, sequencing of various sites in *ompL37* gene, including binding sites and immunogenic epitopes, can be valuable alternatives for future studies.

## INTRODUCTION

*Leptospira interrogans*, causing leptospirosis, is classified in pathogenic spirochetes of the *Leptospira* genus. Leptospirosis, a tropical and zoonotic disease, is typically associated with insufficient sanitation and poverty, which is transmitted mainly through contact with the contaminated urine of reservoir animals (1, 2). Infection caused by *Leptospira* is ranged from unapparent form to fatal liver infection (3). Because of changes in the lipopolysaccharide (LPS) of leptospira, there is a large variety of *Leptospira* serovars, among which over 260 serovars have been identified so far (4). Genetic classification of leptospira with the DNA hybridization method showed 13 pathogenic and 6 saprophytic species in which *L. interrogans* is one of the major causes of leptospirosis (5, 6). The interaction between leptospira and its host depends on the following factors: I) a type of access to host’s body; II) bacterial evasion from the host immune system; and III) adhesion to target tissue by bacterial proteins (7, 8). Accordingly, identification of genetic changes in new proteins, which are important in pathogenesis, leads to the correct understanding of disease and improvement in treatment or prevention such as vaccination (9, 10). Microarray investigations showed that in vitro gene expression of *L. interrogans* is different from it’s *in situ* gene expression. It happens further in genes related to mortality and morbidity (11, 12). Many of these target antigens lie in the leptospiral outer membrane (OM). The OM of pathogenic leptospira spp. contains a number of components including LPS, lipoproteins (including LipL32, LipL21, and LipL41), and phospholipids. The OM proteins are highly conserved across the pathogenic species (13, 14).

It is not completely clear which adhesion proteins can attach to extracellular matrix (ECM) in each step of leptospira growth; nonetheless, some previous studies have reported that OmpL37 is the first protein that can be specifically attached to human skin elastin. Ompl47 is also able to adhere to fibrinogen, fibronectin, and elastin. Nevertheless, OmpL37 has higher adhesion affinity (15). Elastin is present in many body tissues such as blood vessels, skin, intestine, etc. Therefore, leptospira can infect the tissues through OmpL37 (16, 17). Because *ompL37* is expressed only in pathogenic leptospira spp., the expression rate of *ompL37* is higher during infection (18). Accordingly, OmpL37 has an indispensable role in pathogenesis. It is a highly conserved protein among leptospira proteins and exists in the bacterial surface; these properties make OmpL37 a potential candidate for the development of subunit or DNA vaccines. Expression of OmpL37 in pathogenic species and its identification, as a conserved protein, is important reason to design serum diagnostic tests. Moreover, because of higher specificity and lower false positivity, this method is a matter of investigation since it fails to identify saprophytic species.

The cloning and sequencing techniques are the first steps for long term identification and comparison of the gene for development of the vaccine and serum diagnosis tests. The primary objective of this work was to clone and sequence the *ompL37* gene in local and vaccine serovars as well as identification of *ompL37* polymorphism in various serovars to design molecular diagnostic tests in pathogenic and non-pathogenic leptospira.

## MATERIALS AND METHODS

### *Leptospira* serovars and culture

Sixteen *L. interrogans* serovars, including Autumnalis (RTCC 2802), Canicola (RTCC 2805, RTCC 2824, and RTCC 2836), Grippotyphosa (RTCC 2808, RTCC 2825), Hardjo (RTCC 2810, RTCC 2821), Icterohaemorrhagiae (RTCC 2812, RTCC 2823), Pomona (RTCC 2815, RTCC 2822), Serjoe (RTCC 2817), Pyrogenes (RTCC 2835), Australis (RTCC 2840), Bataviae (RTCC 2842) and *L. biflexa* Patoc serovar (RTCC 2819), used in this study were obtained from microbial collections of the Leptospira Reference Laboratory at Razi Vaccine and Serum Research Institute, Karaj, Iran. *L. interrogans* serovars were cultured in EMJH Liquid medium (Difco, Sparks, USA) supplemented with Leptospira enrichment at 30°C (19).

### Extraction of genomic DNA

Genomic DNA was extracted through the standard phenol-chloroform method described by Sambrook and Russell (20). Then, ethidium bromide staining on agarose gel electrophoresis was used to evaluate the extracted DNA, followed by spectrophotometric analysis using the Epoch system (BioTek, New York, USA).

### PCR amplification

A specific primer pair was designed to amplify the 996 bp *ompL37* coding sequence, as shown in Table 1. Then, PCR reaction was performed in 16 μl standard PCR reaction mixture containing 10 pmol of forward primer, 1μl of reveres primer, 2 μl of genomic DNA template and 8 μl of 2 × master mix. PCR parameters in current study were as follow: initial denaturation temperature at 94°C (5 min) followed by 35 cycles of denaturation at 94°C (1 min), annealing temperature at 53°C to 66°C (1 min) and extension at 72°C (1 min). Thereafter, the final extension was carried out at 72°C (10 min). PCR products were analyzed by agarose gel electrophoresis (21).

**Table 1:** PCR primers sequences used to amplify *ompL37* gene

**Primer**	**Oligonucleotide Sequence**	**Primer length (mer)**	**Fragment length**	**Tm°**	**GC%**
F-*ompL37*	5′-AAGGATCCGATAGTCAACTTAG-3′	24	996 bp	56.9	41.7%
R-*ompL37*	5′-TGGGTACCTTAATTTTGTGTTTTT-3′	25		55.9	32%

### PCR product purification

The gels containing DNA fragments were excised using a clean scalpel and placed into a pre-weighed 1.5 ml tube and weighed. The DNA gel extracted fragments were purified using a DNA purification kit (Roche, Germany) via the manufacturer’s instruction.

### Cloning of the *ompL37* gene

The amplified *ompL37* gene was cloned into pTZ57R/T vector according to the manufacturer’s protocol (Thermo Fisher Scientific/USA). Briefly, *E. coli* 10G competent cells were thawed and placed into a pre-cooled 15 ml centrifuge tube. 25 ng of pTZ57R/T vector DNA was added with 100 ng of the purified PCR products and were stirred. Subsequently, the mixture of cells and DNA template were incubated on ice for 30 min. The cells were Heat shocked at 42°C for 45 s, followed by the addition of 960 μl recovery medium and incubated at 37°C in a shaking incubator for 2 h. Finally, the mixture was plated on the LB agar containing ampicillin, IPTG, and X-gal. Then, blue/white colony screening was performed and recombinant colonies were confirmed by colony PCR analysis. Plasmids were extracted from bacterial growth using the DNA plasmid Mini extraction kit (Roche, Germany) according to the manufacturer’s instructions.

### Nucleotide sequencing and homology analysis

Sequences obtained in the current study were compared to those retrieved from the Genbank via the Meg Align software (The software solution for DNA, RNA and protein sequence alignment and analysis).

To determine the phylogenetic relationships of leptospira serovars based on the *ompL37* gene, the phylogenetic tree, similarity and divergence of sequences were drawn using Meg Align software.

## RESULTS

### Cloning of *ompL37* gene

The PCR analysis showed that the amplified 996 bp *ompL37* products belong to pathogenic serovars. These results confirmed the high specificity rate of designed primer pairs used to amplify *ompL37* from these serovars. *E. coli* carrying pTZ57R/T vector with the ligated gene encoding *ompL37* was amplified in a multi cloning site in *lacZ* producing white colonies that did not hydrolyze X-gal by galactosidase when *ompL37* ligated in the *lacZ* region. In contrast, *E. coli* carrying pTZ-57R/T non-ligated gene coding *ompL37* in an MCS produced blue colonies that hydrolyzed X-gal.

### Sequence analysis

**Fig. 1:**
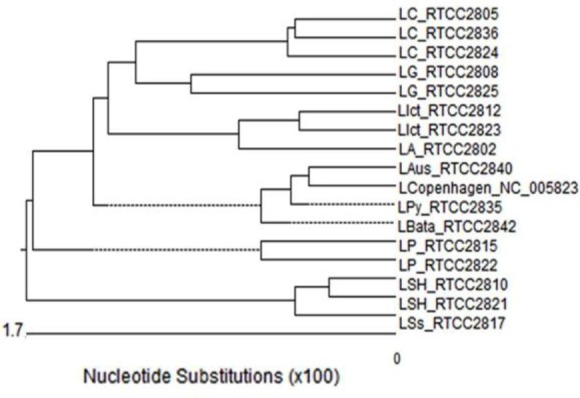
Phylogenetic tree analysis of *ompL37* Gene

In the present research, a total of 16 serovars were sequenced. The *ompL37* gene has been recently identified while only one serotype of *L. interrogans* serovar Copenhagen registered at the Genbank. This serovar was compared with 16 designated serovars in the current work and it was revealed the phylogenetic relationships by considering the single-nucleotide polymorphisms in various serovars and phylogenetic tree (Fig. 1). The comparison of the results, similarity, and divergence of the strains are shown in Table 2.

**Table 2:** Sequence pair distances of *ompL37* gene sequences of different Leptospiral serovars

**Percent Identify**																				

		**1**	**2**	**3**	**4**	**5**	**6**	**7**	**8**	**9**	**10**	**11**	**12**	**13**	**14**	**15**	**16**	**17**		
**Divergence**	1		94.9	91.8	99	85.2	85.1	85.1	85.1	84.9	94.1	93.5	86.3	85	85.7	89.9	90	90.7	1	*L.* Australis
	2	2.1		91.8	95	87.9	87.8	87.8	87.8	87.6	98.8	98.6	89	87.7	85.2	90.3	90.3	90.9	2	*L.* Auturmalis
	3	0.6	1.1		92	92.9	92.8	92.8	92.6	92.3	96.4	90.4	93.7	92.4	94.7	97.7	97.8	98.6	3	*L.* Bataviae
	4	0.8	1.9	0.6		85.4	85.3	85.3	85.2	85	94	93.6	86.4	85.1	86	90.1	90.2	90.9	4	*L. Copenhagen*
	5	1.6	1.7	1	1.6		99.2	99.2	92	91.9	91.8	86.6	93.1	91.9	94	91.4	91.4	91.8	5	*L.* Canicola
	6	1.7	1.8	1.2	1.7	0.9		99.1	92.1	91.7	91.8	86.6	93	91.9	94	91	91.2	91.7	6	*L.* Canicola
	7	1.7	1.8	1.2	1.7	0.9	1		91.9	91.7	91.6	86.5	93	91.8	93.8	91	91	91.7	7	*L.* Canicola
	8	1.7	2.1	1.7	1.8	2.2	2	2.3		98.5	94.5	86.6	98.8	98	98	90.9	90.9	91.5	8	*L.* Grippotyphosa
	9	2	2.4	2	2.1	2.3	2.6	2.6	1.8		94.9	86.3	98.6	97.5	97.7	90.7	90.7	91.3	9	*L.* Grippotyphosa
	10	2.3	1.3	2	2.5	2.2	2.2	2.5	3.2	3.2		99.2	96.3	95	68.3	94.9	95	95.4	10	*L.* Icterohaemorrhagiae
	11	3.7	1.5	2.7	3.4	3.3	3.3	3.4	3.6	4	0.9		87.7	86.7	84	89.1	89.1	89.6	11	*L.* Icterohaemorrhagiae
	12	0.3	0.6	0.3	0.4	0.8	0.9	0.9	1.4	1.7	1.6	2.2		98.7	99	92	92	92.6	12	*L.* Pomona
	13	1.8	2.2	1.8	2	2.3	2.3	2.4	2.5	3	3	3.4	1.6		99	91.4	91.4	98	13	*L.* Pomona
	14	1.2	1.8	1.2	1.2	1.6	1.6	1.8	2.4	2.8	2.5	3.3	1.2	1.2		93.2	93.2	93.5	14	*L.* Pomona
	15	2.9	3	2.5	2.9	3	3.4	3.4	3.6	3.8	3.7	4.4	2.2	3	2.8		99.5	99.1	15	*L.* Serjoe hardjo
	16	2.8	3	2.3	2.8	3	3.3	3.4	3.6	3.8	3.5	4.4	2.2	3	2.8	0.6		99.2	16	*L.* Serjoe hardjo
	17	2	2.2	1.5	2	2.5	2.6	2.6	2.9	3.2	3.1	3.8	1.6	2.5	2.4	1	0.8		17	*L.* Serjoe serjoe
		1	2	3	4	5	6	7	8	9	10	11	12	13	14	15	16	17		

Sequence comparison analysis of 16 native serotypes examined in this study showed that the similarity range among used serovars was 84% to 99.5%, respectively. The results revealed that there was a high similarity among different serovars, indicating that the *ompL37* is a conserved region. Two serotypes of *L. interrogans* serovar including Serjoehardjo (RTCC 2810 and RTCC 2821) had the highest identity up to 95.5% (Table 2). Additionally, two serovars of *L. interrogans* including Serjoe serjoe (RTCC2817) and Serjoare hardjo (RTCC 2821) as well as two serovars of *L. interrogans* canicola (RTCC 2805 and RTCC 2824) showed the homology of up to 92.2%. Two serovars of *L. interrogans* including pyrogenes (RTCC 2835) and Pomona (RTCC 2815) showed a homology of up to 98.7%. Two serovars of *L. interrogans* including Pomona (RTCC 2822) and ICterohaemorrhagiae (RTCC 2823) had the lowest identity of about 84%.

## DISCUSSION

Leptospirosis as an important infection caused by *L. interogans* is able to transmit from livestock to humans (22). This bacterium is classified into pathogenic and non-pathogenic species which are apparently similar but different conditions and characteristics required for growth and performance in the host (7, 8). Bacterial adhesion proteins are responsible for attaching to the cell surface and colonizing host tissues; thus, these proteins play a determinant role in bacterial pathogenicity (16). *ompL37* attacks to the skin elastin as well as to elastin of lungs and blood vessels and is known to play a role in pathogenesis in the host. The OmpL37 is present on the surface of pathogenic Leptospira spp. and its sequence is well conserved. It serves as a cell adhesion and plays a remarkable role in the pathogenicity. Therefore, it can be used in diagnostic tests and vaccine producing procedure (23, 24). The major limitation in the development of a universal vaccine is related to the absence of cross-immunity among all *Leptospira* species (25). The *Leptospira* vaccine is still made from the lethal strain. The results of the present study were in utmost of importance, because of the revealing conserved sequence. The identification of conserved sequences makes it an appropriate choice among *Leptospira* pathogenic species for the development of a recombinant vaccine with cross immunity among different strains. A diligent study in 2015 has reported that OmpL37 is potentially a surface-exposed outer membrane protein, expressed during the infection, and able to induce the immunity responses strongly. However, OmpL37 when presented as a recombinant protein in Alhydrogel and DNA vaccine preparations, has not enough effectiveness to stimulate protection against leptospirosis (10). Another study showed that antibody against OmpL37 is mostly caused the augmentation in adhesion affinity of OmpL37 to host elastin instead of immunity against *Leptospira* (15).

On the other hand, some investigations have mentioned that OmpL37 is an important pathogenicity factor in the incidence of leptospirosis (22). Accordingly, it is imperative to identify all binding sites and antigenic regions in the OmpL37 protein. Identifying *ompL37* gene sequence homology in all serovars for their classification is in utmost of importance. The hypothesis that “the adhesion sites are different from antigenic regions” is significant point because making recombinant peptides from antigenic epitopes can lead to developing universal vaccine production. Because of the first investigation in this scope, there was no way to compare *ompL37* gene sequence of various serovars.

As it might to be expected, the similarity in sequences was up to 95.5%; this information could be used to develop diagnostic kits and produce antibodies for researches. Additionally, these sequences can be used to develop OmpL37 recombinant protein. Diagnosis of leptospirosis in early stages is important since it brings the timely treatment of patients and prevents the disease to become acute. It is worth noting that diagnosis of *Leptospira* standard based on microscope agglutination test (MAT) is faced with two challenges: 1- Identification of antibody by live bacteria may expose laboratory personnel to the hazardous organism. 2- Raising antibody titer to the identification stage is a time-consuming process (at least one week). Since the MAT test is designed based on the antibody, diagnosis of diseases would be delayed. Designing molecular tests based on the conserved genes including *ompL37* can be lead to early diagnosis and identification of pathogenic species.

This work reported that the *ompL37* gene represented a high similarity among *L. leptospira* pathogenic serovars. The results also confirmed that sequences of *ompL37* gene can be used to improve vaccine and diagnostic tests. Furthermore, sequencing of various sites in *ompL37* gene including binding sites and immunogenic epitopes would help future studies.
